# Outdoor access versus conventional broiler chicken production: Updated review of animal welfare, food safety, and meat quality

**DOI:** 10.1016/j.psj.2025.104906

**Published:** 2025-02-17

**Authors:** Yan L. Campbell, Lin L. Walker, Brooke M. Bartz, James O. Eckberg, Allison N. Pullin

**Affiliations:** a Prestage Department of Poultry Science, North Carolina State University, Raleigh, NC 27695, USA; b General Mills Inc., Golden Valley, MN 55426, USA

**Keywords:** Animal welfare, Broiler, Food safety, Meat quality, Outdoor access

## Abstract

Growing consumer demand for animal welfare and environmental sustainability in the poultry industry is driving the adoption of outdoor access for broiler chickens in the United States. However, shifting to outdoor access from conventional housing may pose tradeoffs for animal welfare, meat quality, and food safety. Research comparing conventional and outdoor access housing on these attributes has not been reviewed for approximately a decade. We reviewed and compared animal welfare, food safety, and meat quality outcomes in conventional versus outdoor access broiler production, focusing on recent research. Despite the prevailing notion that outdoor access improves animal welfare due to more behavioral opportunities, the utilization of the range is highly variable and affected by a variety of environmental, management, and bird characteristics. Outdoor areas containing vegetation and tree cover promote use by the birds, and slow-growing breeds appear to be best suited for these production systems. Typically, welfare-related health outcomes (i.e., footpad dermatitis, mortality, and lameness) are improved with outdoor access. However, birds with outdoor access are at a higher risk for endo- and ectoparasitic infections. Antimicrobial resistance is typically lower on outdoor access farms, and birds with outdoor access have more diverse microbiomes. There are mixed results for the prevalences of *Salmonella* and *Campylobacter* between conventional and outdoor access farms. Meat quality varies in complex ways related to rearing system, age, breed, diet, and behavior. Meat from outdoor access broilers may present better taste or flavor, yet there can be tradeoffs for texture and moisture, particularly for older, slower-growing breeds that are typical of outdoor access production. Taken together, studies to date indicate multiple benefits and tradeoffs for animal welfare, food safety, and meat quality. Variations in management between farms and certification criteria result in inconsistent outcomes. The majority of outdoor access research has been conducted outside of the United States. Region-specific research accounting for geography, climate, and available breeds would be beneficial for improving outdoor access production outcomes in the United States.

## Introduction

The United States’ broiler chicken industry is developing production guidelines and practices to meet growing demands for improved animal welfare while delivering a product with high meat quality and safety at an affordable price. Recent emphasis has been focused on developing production systems that provide some level of outdoor access to improve health and support behavioral opportunities (Elkhoraibi et al., 2017; Rothrock et al., 2019). This represents a significant shift in the industry for American broiler chicken production, which has predominantly housed birds exclusively indoors and followed the welfare guidelines established by the National Chicken Council (NCC, 2022).

Providing outdoor access offers poultry more behavioral opportunities, but it also comes with its own unique management challenges compared to conventional housing. Outdoor access exposes poultry to increased disease and mortality risks (e.g., predation, parasites, pathogens), and breeds that thrive in conventional housing may not be well adapted for outdoor access (van de Weerd et al., 2009; Sossidou et al., 2011). However, there are management strategies to improve poultry health and welfare in outdoor systems (Sossidou et al., 2015). Furthermore, some nutritive and sensory qualities of poultry meat are reported to improve when birds have outdoor access (Sossidou et al., 2015). There are also possible economic benefits for American poultry farmers with pasture-raised poultry supporting premium pricing: the September 2024 average retail price for a pasture-raised boneless breast was $14.13/lb compared to $2.71/lb for conventional boneless breast (USDA AMS, 2024).

Reviews about poultry welfare and meat quality in outdoor access housing were published approximately a decade ago (van de Weerd et al., 2009; Sossidou et al., 2011, 2015). Considerable research has since been published about poultry welfare, meat quality, and food safety in outdoor access systems. However, minimal research has directly compared conventional and outdoor access housing systems to one another, particularly in the United States. Region-specific research is valuable as geography, climate, and available breeds affect animal welfare, food safety, and meat quality outcomes, which are critical for the sustainability of production systems. By providing an updated review of outdoor access literature that also considers these interactions, we may inform certification programs and initiatives in the United States on the long-term feasibility and tradeoffs for producers to switch to outdoor access. Therefore, for the current review, the main objective was to summarize key findings comparing conventional and outdoor access housing in animal welfare, food safety, and meat quality, particularly incorporating research published since previous reviews. Additionally, the location where research was conducted and the breeds used are highlighted as factors that introduce variability and mediate research outcomes, a theme that is explored to guide future work in the United States.

## Current standards and definitions

In the United States, outdoor access broiler housing includes United States Department of Agriculture (USDA) organic, free-range, and pasture-raised broiler production systems. USDA organic requires that animals must be provided with access to the outdoors, but the amount of space, access time, or quality of the outdoor area are currently not specified (USDA AMS, n.d.). However, the USDA recently announced that it will require 1 ft^2^ (0.09 m^2^) of outdoor space for every 5 lbs (2.27 kg) of bird in the flock, or at least 2 ft^2^ (0.19 m^2^) of outdoor space per broiler by January 2, 2029 (USDA-AMS, 2023). Broilers will be able to be confined for the first 4 weeks of life to maintain optimal brooding conditions (USDA-AMS, 2023).

The USDA does not regulate the definition of free-range and until recently did not regulate the definition of pasture-raised, so the criteria for these management systems has been dependent upon third-party animal welfare certification and audit programs. Certification programs differ in some of the criteria for each definition, which have been summarized in Table 1. Free-range is commonly defined as birds having access to an outdoor range area (e.g., 0.19 m^2^/bird for Certified Humane) that has some vegetation and overhead cover (Table 1). Birds have free choice to enter and exit the range from doors on the poultry house for at least eight hours per day, and the birds are confined in the house at night for predator protection (Table 1). However, conditions (e.g., inclement weather, predation risk, range condition) and number of days that birds are able to access the range are not specified in most audit guidelines, resulting in a potentially high level of variation between farms as to how much outdoor access the birds actually receive.

**Table 1:** Comparison of space requirements, rearing programs, and outdoor access for poultry across certification systems.

Criteria	Certification system			
	American Humane^1^	Certified Humane^2^	Global Animal Partnership^3^	National Chicken Council^4^
**Indoor Space Requirements**	Free-range: 34 kg/m²	Free-range: 30 kg/m² Pasture-raised: 24.4 kg/m² (mobile coops)	Step 3: 29 kg/m² Step 4-5+: 27 kg/m²	Conventional: 31.7 kg/m² (<2.0 kg) 36.6 kg/m² (2.0-2.5 kg) 41.5 kg/m² (2.51-3.4 kg) 43.9 kg/m² (>3.4 kg)
**Outdoor Space Requirements**	Free-range: Not specified	Free-range: 0.19 m²/bird Pasture-raised: 10 m²/bird	Step 3: Outdoor area ≥ 75 % of indoor floor space Step 4: ≥ 100 % of indoor floor space	Conventional: N/A
**Rearing program**	Free-range: Outdoor access by 35 days of age	Free-range & Pasture-raised: Outdoor access by 28 days of age	Steps 3-5+: Outdoor access by 28 days of age	Conventional: All stages of production are indoors
**Predator protection**	Free-range: Enclosed in the fixed house or mobile shelter at night	Free-range & Pasture-raised: Enclosed in the fixed house or mobile shelter at night	Steps 3-5+: Birds must be enclosed in the fixed house or mobile shelter at night	Conventional: Confined indoors at all times
**Outdoor Access Timing**	Free-range: Minimum 8 h/day, except for situations with high risk of avian influenza or other highly pathogenic diseases	Free-range: Minimum 8 h/day; except for situations with disease, higher-than-average mortality due to predation, or extreme weather. Pasture-raised: Minimum 6 h/day every day for 12 months per year. In an emergency, birds may be confined in fixed or mobile housing 24 h per day for no more than 14 consecutive days	Steps 3-5+: Any chickens slaughtered before 28 days of age must have access to the outdoors during daylight hours for a minimum of 2 weeks Step 3: Continuous outdoor access during daylight hours unless climatic conditions pose a welfare risk Step 4: Continuous access to pasture during daylight hours. If the risk from climatic conditions is considered extreme (e.g. heavy precipitation, tornadoes, hurricanes, monsoons, blizzards, floods or non-typical weather for the season such as large swings in temperature), access to outdoor areas may be restricted as long as the restriction does not exceed 25 days (cumulative total) throughout a calendar year (January 1-December 31) Step 5 and 5+: Continuous access to pasture during daylight hours. If the risk from climatic onditions is considered extreme, access to outdoor areas may be restricted as long as the restriction does not exceed 5 consecutive days and 25 days (cumulative total) throughout a calendar year	Conventional: N/A
**Range Conditions**	Free-range: Feed and drinking water evenly distributed throughout the outdoor area Active management of range (e.g., rotation, reseeding, drainage) Partial overhead cover; sufficiently large, shaded area in the summer	Range must consist mainly of living vegetation; also coarse grit must be available Active management of range (e.g., minimize heavily worn areas, minimize build-up of disease-causing agents) Shade distributed throughout range; shaded area must accommodate 30 % of the birds at one time	Steps 3-5+: Provisions must be provided to encourage chickens to range (e.g., bushes, shrubs, shade cloth, A-frame structures, and perches) and not attached to the house; must start within 4.5 m of the house; must provide a cumulative total of at least 0.75 m^2^ of cover for every 100 chickens in the flock Step 3: At least 25 % of each occupied outdoor area must be covered with vegetation and/or forage Step 4: Within 61 m from the house, at least 50 % of each occupied pasture area must be covered with vegetation and/or forage accessible at chicken height Step 5 and 5+: Within 61 m from the house, at least 75 % of each occupied pasture area must be covered with vegetation and/or forage accessible at chicken height; denuded areas cannot extend more than 10 ft (3m) from the house	Conventional: N/A

1 American Humane Certified (2019)

2 Certified Humane (2022)

3 Global Animal Partnership (2020)

4 National Chicken Council (2022)

The USDA recently announced that pasture-raised claims must be supported with written documentation that animals are raised on land covered mostly with vegetation for the majority of their lives (Food Safety and Inspection Service, 2024). Certified Humane, a third-party certification program, broadly differentiates pasture-raised from free-range as pasture-raised birds having guaranteed daily exposure to a larger outdoor pasture area (10 m^2^/bird) that is covered with vegetation (Table 1). Birds in this program have outdoor access for all 12 months of the year and cannot be indoors for more than 14 consecutive days, assuring more consistent outdoor access than free-range (Table 1). The American Pastured Poultry Producers Association aims to publish management standards for third-party pastured poultry audits in partnership with the American Grassfed Association, where birds will be required to live on pasture for a majority of their lives in a mobile coop (APPPA, 2024). Finally, silvopasture is an emerging type of outdoor access for poultry that is currently not distinguished in certification programs. Silvopasture is an agroforestry approach to integrate animal production with tree production (Jacobs, 2024), but the management guidelines for birds in this type of outdoor access system are undefined.

## Animal welfare

The amount and quality of space provided to birds inherently affects their abilities to perform highly motivated behaviors and access key resources, such as feed and water. Stocking densities vary widely according to the rearing program and have been summarized in Table 1. Indoor exclusive rearing includes a maximum of 31.7 to 43.9 kg/m^2^ recommended by the National Chicken Council, depending on the target market weight for the flock, whereas birds with outdoor access have a lower indoor stocking density (29.3 to 34.2 kg/m^2^) depending on the certification program. The latter are also allotted additional space to access outdoors, which again varies greatly from 0.19 m^2^/bird for free-range and up to 10 m^2^/bird for pasture-raised. Although these birds are given access to the outdoors, there are environmental factors that must be taken into consideration for when and how long birds will be able to access this outdoor environment. During brooding periods, disease and predation risks, and inclement weather, birds may benefit by staying indoors, as indicated by some level of acceptable confinement that is included in each certification program. We compared indoor exclusive housing to outdoor access housing for broilers using the framework for animal welfare as proposed by Fraser et al. (1997) to provide an updated literature review evaluating: 1) opportunities to express natural behaviors, 2) biological health and functioning, and 3) affective state.

### *Natural behaviors*

Highly motivated behaviors of chickens include dustbathing, foraging, perching, and social behaviors, although these were primarily established in laying hens that differ in age, genetics, and body conformation from broiler chickens (Weeks and Nicol, 2006). Broiler chickens generally spend at least 50 % of the day sitting and inactive, and they typically become less active as they age by sitting for as much as 70 to 80 % of the day (Ross 308 broilers, Denmark, Bach et al., 2019; 14 broiler breeds differing in growth rate, Canada, Dawson et al., 2021). In exclusively indoor housing, birds may perform dustbathing and foraging behaviors if they can manipulate litter bedding that is loose, friable, and without excessive moisture (i.e., no caking or clumping). If environmental enrichment is provided in indoor housing, other behaviors may be promoted, but these were extensively reviewed in two recent publications (Riber et al., 2018; Jacobs et al., 2023) and therefore indoor-housing enrichment will not be included in the focus of the current review.

If birds are provided with outdoor access, then additional space is available for birds to utilize for behavioral opportunities. Walking and foraging behaviors are most common in the outdoor access area, while birds spend more time eating, drinking, and lying down in their indoor structures (Delaware slow-growing breed, Arkansas, United States, Fanatico et al., 2016). The average percentage of the flock observed on the outdoor range varies between studies (e.g., 13 % in Fanatico et al., 2016 versus 62 % in Castellini et al., 2016 for the Ancana slow-growing breed, Italy), which is linked to environmental, management, and bird factors, as well as behavioral sampling methodology.

Season, temperature, time of day, and resources available on the range contribute to large variability in ranging behavior (Jones et al., 2007). Rain, radiation, and wind result in fewer chickens outside (Sasso T451 slow-growing breed, Belgium, Stadig et al., 2017). Fewer birds were observed on the range in the afternoon, particularly in the summer, during the hottest and brightest time of day (Sherwood White slow-growing breed, United Kingdom, Dawkins et al., 2003; 6 % in the afternoon compared to 20 % in the morning, Rowan Ranger and HubbardJA57/HubbardJA87 slow-growing breeds, Sweden, Göransson et al., 2021). However, birds ranged less in the winter compared to spring and summer seasons, which may be due to temperature but also less overhead cover from trees that have fewer leaves in the winter season (Sherwood White slow-growing breed, United Kingdom, Dawkins et al., 2003). Similarly, Taylor et al. (2017a) reported 10.6 % and 36.7 % of the flock utilized the outdoor range in the winter and summer seasons, respectively (Ross 308 fast-growing breed, Australia).

Chickens prefer range habitats with trees, which provide shade, dry areas for dustbathing, and protection from aerial predators (Dawkins et al., 2003). Providing vegetative coverage that is tall enough to provide shade (e.g., bushes, trees, and tall grasses) results in more broilers utilizing outdoor space and venturing further than when they are provided with artificial shade structures (e.g., wooden panels, A-frames, camouflage nets; Sasso T451 slow-growing breed, Belgium, Stadig et al., 2017). Legume species in the outdoor range resulted in Marshall fast-growing broilers displaying more locomotor activity and comfort behaviors (i.e., dustbathing, preening) compared to those housed indoor-only or with outdoor access that did not contain legume species, which may be related to grazing opportunities and/or ground condition (Nigeria, Oke et al., 2021). In total, there are numerous management factors that influence a flock's use of outdoor space, such as the age when outdoor access was first provided, the timing of outdoor access within a day (i.e., time of day doors are opened and closed, duration of time that doors are open), the design of exits/pop-holes to access the range (including ramps), maximum ranging distance allowed, flock size, type of range resources present (e.g., shade structures, feed and water on range), vegetation density and species, and orientation of shelter to sunlight and shade (Sherwood White slow-growing breed and Ross 308 fast-growing breed, United Kingdom, Jones et al., 2007; Ross 308 fast-growing breed, Australia, Taylor et al., 2020).

In addition to the environmental and management conditions, bird characteristics influence range use variability. Birds from slow- and medium-growing breeds spent a greater amount of time utilizing outdoor range compared to fast-growing breeds (slow, Ancona, Leghorn, and Cornish x Leghorn: 56 to 62 %; medium, Gaina, Robusta Maculata, Kabir, and Naked Neck: 42 to 49 %; fast, Ross 308: 19 % of time observed; Italy, Castellini et al., 2016). Using a global positioning system (**GPS**), an Ancona slow-growing breed traversed 10 times the daily distance of range (1,230 m) than a Ross 308 fast-growing breed (125 m; Italy, Dal Bosco et al., 2010). Behavior observations confirmed that the Ancona slow-growing breed spent more time in locomotion and foraging on the range than the Ross 308 fast-growing breed (Dal Bosco et al., 2010). Indeed, even in an indoor-only environment, most unspecified slow-growing breeds were more active and utilized enrichment more than unspecified fast-growing breeds at younger ages (4 and 5 weeks of age; Canada, Dawson et al., 2021). Similarly, a Hubbard JA57 slow-growing breed spent more time standing and locomoting than a Ross PM3 fast-growing breed in both indoor-only and outdoor access housing systems, while the Ross PM3 fast-growing breed spent more time feeding, drinking, and dustbathing (Turkey, Abdourhamane and Petek, 2023).

Individual bird characteristics within a breed can also influence use of outdoor space within a flock. Using radio frequency identification (**RFID**) to track individual bird range use, the percentage of birds in the flock that accessed the range at least once was 32.8 % and 87.3 % in winter and summer, respectively (Ross 308 fast-growing breed, Australia, Taylor et al., 2017b). These findings for individual bird range use are higher than when the same researchers recorded behavior at the flock level (10.6 % and 36.7 % of the flock utilizing the range in the winter and summer, respectively; Taylor et al., 2017a). These results highlight the limitation of flock-level behavior observations in assuming that the percentage of birds on the range are always the same individuals. Individual tracking reveals that, when given the choice of outdoor access, the majority of birds will choose to access the range at least once in certain conditions (Taylor et al., 2017b).

Within ranging birds, there are individuals that travel further distances and use the range more than birds that ranged within a closer proximity to the house (Ross 308 fast-growing breed, Australia, Taylor et al., 2020). The distance rangers weighed less and had better gait scores for mobility than the closer rangers, again suggesting that individual physical characteristics and welfare indicators influence birds use of the range (Ross 308 fast-growing breed, Australia, Taylor et al., 2020). Foraging behavior in early life (i.e., in an indoor rearing environment for the first 5 weeks) positively correlated with outdoor range use during early range access (5 to 7 weeks of age) and late range access (9 to 12 weeks of age) in a Naked Neck slow-growing breed (France, Ferreira et al., 2022). Individual Brown Nick laying hens that utilized an outdoor range more had higher levels of proliferating cell nuclear antigen expression in the rostral hippocampus, which is a marker of hippocampal neurogenesis that is associated with behavioral plasticity (Switzerland, Armstrong et al., 2020). These findings suggest a possible relationship between exercise, bird's mental map of their home range for navigating to resources, and/or personality (Armstrong et al., 2020). Therefore, variation in underlying individual behavior and neurological traits mediate individual bird range use, in addition to the environmental, management, and breed characteristics.

### *Biological health and functioning*

Mobility, footpad and hock joint dermatitis, thermal stress, endo- and ectoparasites, and mortality are key health indicators providing insights into the welfare status of broiler chickens, regardless of housing environment (Manning et al., 2007). Conditions that result in birds having prolonged contact with high litter moisture content can lead to inflamed, necrotic footpad and hock tissue, which is painful and contributes to impaired mobility (i.e., lameness; Bessei, 2006). At higher stocking densities, birds are unable to dissipate metabolic heat as effectively and experience higher litter moisture due to higher concentrations of manure (Bessei, 2006). Consequently, birds housed at higher stocking densities are at a higher risk of heat stress, reduced growth rates, and a higher prevalence of dirty feathers and footpad dermatitis (Bessei, 2006).

Outdoor access can be associated with improved health. In Brazil, conventional indoor-only housing with a Cobb 500 fast-growing breed scored lower than free-range housing with a Label Rouge slow-growing breed on several animal-based welfare outcomes, demonstrating higher prevalences of dirty feathers, panting, moderate to severe lameness, hock joint burns, and mortality in indoor-only housing (Sans et al., 2023). Indoor-only housing outperformed free-range housing on a few resource metrics, such as litter quality and dust, which is likely due to having more controlled ventilation in an indoor-only house (Sans et al., 2023). A “Total Welfare Score” was developed for production systems in the Netherlands by comparing animal-based health measures (i.e., footpad and hock joint dermatitis, breast blisters, scratches and wounds, and mortality) and resource-based measures (i.e., stocking density, early feeding, environmental enrichment) across three types of commercial broiler production systems (de Jong et al., 2022). The three types of production differed in their housing design, stocking density, and breed (only identified by maximum growth rate), but generally the two production systems with lower stocking densities, slower growth, environmental enrichment, and/or outdoor access had higher Total Welfare Scores than conventional indoor-only housing (de Jong et al., 2022). However, when including only animal-based health measures in the Total Welfare Score, the researchers demonstrated a wide range of scores within each production system (de Jong et al., 2022).

Regardless of the category of production system, individual farm management contributes to variation in animal-based health measures. Similarly, interviews with organic broiler farmers in Sweden (including outdoor access) revealed high variability between farms for flock management (Göransson et al., 2020). Farmers tailored management practices to characteristics of their individual farms, where management variation between farms is likely also contributing to inconsistent animal welfare and production outcomes for farms categorized as the same production type (i.e., organic; Göransson et al., 2020). When the Welfare Quality® Protocol was used to evaluate commercial free-range broiler farms in Brazil, footpad dermatitis was highly variable and linked to range quality differences between sites, such as caked soil, lack of shelter and vegetation, and no implementation of rotational grazing (Label Rouge slow-growing breed, Sans et al., 2014).

Within an outdoor access flock, birds that utilize the range also have improved health. Researchers identified birds with the most outdoor range visits (outdoor-preferring), the least outdoor range visits (indoor-preferring), and median outdoor range visits (outdoor-moderate; Sasso and Green-legged Partridge slow-growing breeds, Poland, Marchewka et al., 2020). Indoor-preferring birds were more likely to have respiratory infections and toe injuries, but it is unclear if the indoor environment per se created these welfare concerns (e.g., more exposure to dust and/or poor litter quality) or if birds with a lower welfare status do not utilize the outdoor range as frequently (Marchewka et al., 2020). Compared to birds that never went outside, ranging birds had cleaner vents, lower ascites indexes, and lower prevalence of pericardial fluid, suggesting that ranging birds have better health that may promote their ability to utilize the range (Ross 308 fast-growing breed, Australia, Taylor et al., 2018). Similarly, broilers that were evaluated for mobility on the outdoor range had better gait scores (56 % with no gait issues) compared to broilers evaluated in the indoor barn (23 % with no gait issues), indicating that range use either improves mobility or birds with better mobility are more likely to use the range (Rowan Ranger and HubbardJA57/HubbardJA87 slow-growing breeds, Sweden, Göransson et al., 2021). Greater locomotor activity and possibly ingesting grit on the outdoor range resulted in improved bone health (i.e., 37.9 % higher tibia bone mineral content and 15.4 % higher tibia bone mineral density) for broilers with outdoor access compared to broilers housed only indoors (Freedom Ranger slow-growing breed, Iowa, United States, Elmore et al., 2023).

Endo- and ectoparasitic infections are associated with reduced growth rates, poor feather condition, and increased mortality (Jeni et al., 2021). In laying hens, ectoparasitic Northern fowl mite infestations were also associated with increased hen preening behavior and skin lesions on infested birds, indicating discomfort and injuries negatively affecting bird welfare (Murillo et al., 2020). A relatively recent article reviewed the prevalence of gastrointestinal parasites (i.e., coccidia, nematodes, cestodes) and ectoparasites (i.e., red mites and lice) in outdoor access production for broilers and laying hens (Jeni et al., 2021). In brief, endo- and ectoparasitic infections are health risks in both indoor-only and outdoor access environments, but birds with outdoor access tend to be at higher risk than indoor-only birds, depending on how the environment is managed. Similarly, a meta-analysis and systematic literature review on the global prevalence of endoparasitic helminth infections in chickens (including laying hens and broilers) found that free-range and backyard systems had a higher pooled prevalence of helminth infections (82.6 and 84.8 %, respectively) than those reared in indoor-only production systems (71.3 % for deep litter systems; Shifaw et al., 2021). In Brazil, commercial broiler breeds with outdoor access during the daytime were infected with 146.2 ± 125.1 counts of helminths per bird, while no helminths were counted in indoor-only commercial broiler breeds (da Silva et al., 2018). In Lebanon, mixed mite-helminth infestations were found in 100 % of 17 semi-open broiler farms with outdoor access and only 50 % of six closed broiler farms (Shaib and Obeid, 2022). In the United States, a broad diversity of endo- and ectoparasitic species have been identified in outdoor access poultry production, suggesting that a variety of management strategies should be considered to mitigate these infections (Alabama, Carrisosa et al., 2021; Georgia, Terra et al., 2021; California, Idaho, Oregon, and Washington, Chambless et al., 2022 and Cornell et al., 2022). Coccidia in particular was highest in broilers during the first week of pasture access and also in the spring compared to the summer and winter seasons (Georgia, Terra et al., 2021). Taken together, these findings indicate that birds’ risk of infection is substantially greater for outdoor access, and the magnitude of the differences may vary with bird age, climate, and geographic region.

Other health outcomes in outdoor environments can also be highly variable and confounded by breed, range quality, and thermal conditions. Many studies cited in this paper could not disentangle the effects of bird characteristics versus environment-mediated health outcomes. Slow-growing breeds (i.e., Naked Neck, Kabir, Ancona, Leghorn, Cornish x Leghorn, Gaina, Robusta Maculata, Hubbard JA57) have lower mortality, improved feather condition, and lower prevalence of footpad and hock joint dermatitis, breast blisters, and lameness compared to fast-growing breeds (i.e., Ross 308 and Ross PM3; Italy, Dal Bosco et al., 2014; Italy, Castellini et al., 2016; Turkey, Aksoy et al., 2021a; Turkey, Abdourhamane and Petek, 2023; Turkey, Abdourhamane and Petek, 2024). However, even within genotypes classified broadly as slow-growing, there are differences in adaptability to the outdoor range, such that some slow-growing breeds (i.e., Hubbard CY5XJA87 and Hubbard M22XJA87) are less active and have higher rates of mortality, footpad dermatitis, breast blisters, and poor feather condition than other slow-growing breeds (i.e., Hubbard RedJA and Rowan Ranger, Italy, Mancinelli et al., 2020). These findings indicate that there are breed-specific effects on behavior and adaptability to outdoor systems beyond growth rate alone.

The type, density, and height of vegetation available on the outdoor range can influence the ground quality, particularly moisture. In paddocks containing tree cover (i.e., silvopasture), the ground may stay dryer than open pastures. Indeed, researchers found a lower prevalence of footpad dermatitis in Ross 708 fast-growing broilers with access to silvopasture compared to Ross 708 fast-growing broilers with access to open pasture (Virginia, United States, Paneru et al., 2023). Another study found legume species on range to promote locomotor activity and reduce lameness for Marshall fast-growing broilers compared to the same breed housed only indoors or in a range paddock with only grasses (Nigeria, Oke et al., 2021). However, another trial found no differences for footpad or hock joint dermatitis for Hubbard ISA Red JA slow-growing broilers housed on different species of grasses (Turkey, Bashir et al., 2023).

Heat stress is a significant health concern in broiler production, regardless of housing system, and is particularly exacerbated in the summer months. For indoor-only and outdoor access housing in the summer, mortality increased and growth rate decreased to a greater extent for Ross 308 fast-growing broilers than Hubbard JA57 slow-growing broilers because of heat stress (Turkey, Aksoy et al., 2021a). Research comparing fixed housing versus hoop structures with and without outdoor access found reduced growth rates in fast-growing Cobb broilers housed in hoop structures, where minimal ventilation and excessive heat created significant heat stress for the birds (Arkansas, United States, Moyle et al., 2014). However, outdoor access alone did not negatively affect growth rate, as broilers from fixed housing with and without outdoor access achieved similar growth rates (Moyle et al., 2014). When comparing physiological indicators of immunity and stress between indoor-only or pastured broilers, indoor-only broilers displayed higher eosinophil counts than pastured broilers, suggesting a higher inflammatory response that the authors linked with heat stress (Cornish Rock fast-growing breed, Alabama, United States, Liles et al., 2015). Other immunity metrics, such as lymphoid organ weight and counts of other leukocyte cells, did not differ between housing environments (Liles et al., 2015).

Incorporating pre-existing vegetive borders (i.e., hedgerows) into the range area is a strategy to improve animal health and production efficiency by providing shade and shelter to mitigate heat stress, as well as promoting range use and reducing visibility to predators (Delgadillo et al., 2021). The slow-growing Red Ranger breed showed better immune functioning when provided with hedgerow access (indicated by elevated bacterial killing ability and lower hemodilution) compared to the same breed without hedgerow, possibly from ingesting immunomodulatory agents from the hedgerow area during foraging (Oregon, United States, Delgadillo et al., 2021). The fast-growing Cornish Cross breed had a higher average weekly gain with access to hedgerow compared to the same breed without access, which authors attributed to a reduction in heat stress (Oregon, United States, Delgadillo et al., 2021). Providing artificial structures on the range for heat abatement, such as screened shelters or overhead shade panels, also promoted use of an outdoor range by Delaware slow-growing broiler chickens (Arkansas, United States, Fanatico et al., 2016).

### *Affective state*

Environmental complexity and novelty improve the affective state of broilers, which can be provided with outdoor access (Riber et al., 2018; Jacobs et al., 2023). Research on the relationship between outdoor access on broiler affective state specifically is limited and primarily focused on measuring fearfulness with a tonic immobility test and physiological markers of stress. Broilers with outdoor access did not differ in their fearfulness responses (Hubbard JA57 slow-growing and Ross 308 fast-growing breeds, Turkey, Abdourhamane and Petek, 2024) or heterophil:lymphocyte ratios (Cornish Rock fast-growing breed, Alabama, United States, Liles et al., 2015) compared to broilers housed only indoors. Pastured birds initially showed a decrease in the heterophil:lymphocyte ratio after two weeks of outdoor access compared to their early-life indoor rearing ratios, indicating that outdoor access reduced stress (Liles et al., 2015). However, their heterophil:lymphocyte ratios increased during the last two weeks on pasture, which the authors related to heat stress (Liles et al., 2015). Similarly, another study reported that Marshall fast-growing broilers with outdoor access were more fearful than Marshall fast-growing broilers housed only indoors, which may be caused by exposure to a greater variety of stressors outside (e.g., higher predation risk, greater distance from flock mates and resources, and thermal stress; Nigeria, Oke et al., 2021). Indeed, individual Brown Nick laying hens that utilized an outdoor range more than others had lower expressions of doublecortin in the caudal hippocampus, which is a neurological marker pattern indicative of higher stress levels (Switzerland, Armstrong et al., 2020).

The mixed findings of fearfulness and its relationship to outdoor access are likely confounded by other factors, such as breed and range quality. Slow-growing broilers (i.e., Ancona, Leghorn, Cornish x Leghorn, Robusta Maculata, Naked Neck, Hubbard JA57) are generally less fearful than fast-growing broilers (i.e., Ross 308; Italy, Castellini et al., 2016; Turkey, Abdourhamane and Petek, 2024). Outdoor access studies commonly use different breeds, making it difficult to generalize the relationship between affective state and production system among studies. Some vegetation species on the range resulted in more pronounced fear responses than others for Marshall fast-growing broilers (Nigeria, Oke et al., 2021). However, another study reported that pasture vegetation species does not affect fearfulness for Hubbard ISA Red JA slow-growing broilers (Turkey, Bashir et al., 2023). Height and density of vegetation may have a more consistent effect on fearfulness than species alone. For example, when tree cover is available in the outdoor range (i.e., silvopasture), Ross 708 fast-growing broilers are less fearful than broilers of the same breed reared in an open pasture (Virginia, United States, Paneru et al., 2023). As a result, comparing affective state measures between studies is also challenging when range quality and vegetation structure varies widely among studies.

Taken together, these studies report mixed findings that outdoor access may be stressful or cause fear. Studies are limited and confounded when compared. Some indicators of positive affective state (e.g., play, cognitive bias, motivation, preference) have not been directly measured. Preference for using outdoor access can be loosely evaluated by comparing the proportion of birds that are inside a shelter versus outside on the range. Although it is not a formal preference test, this distribution could be an indicator of birds choosing to utilize the environment they prefer. Preference and choice/control in an environment are assumed to result in positive affective states (Englund and Cronin, 2023; Jacobs et al., 2023), so having the choice of outdoor access likely benefits the affective state of individual birds.

Broadly, slow-growing breeds appear to benefit from outdoor access more than fast-growing breeds due to greater outdoor space use, improved health outcomes, and less fearfulness. Outdoor areas with living vegetation, including tree cover, promoted use of the space by broiler chickens. However, highly variable management practices between farms and environmental factors (e.g., weather, geography, and endo- and ectoparasite prevalence) can also lead to highly variable animal-based welfare outcomes. Emphasis on monitoring and improving animal-based welfare outcomes in outdoor access production may result in more consistent animal welfare in this production system.

## Microbial food safety

Mixed findings have been reported on food safety when comparing conventional and outdoor housing environments for broilers. In some cases, outdoor access systems have shown fewer foodborne pathogens than broilers in conventional systems, despite difficulties in maintaining high biosecurity under natural exposure to outdoor elements, wildlife, and limited interventions to reduce pathogens. The top two foodborne pathogens in poultry, *Salmonella* and *Campylobacter*, are common inhabitants of the gastrointestinal tract of poultry and have been summarized by prevalence and production type, as well as antibiotic resistance from various studies in Table 2. Several studies reviewed for microbial food safety did not specify the breed or growth rate of the birds sampled in their methodology, so bird characteristics are only mentioned for studies that did include this information.

**Table 2:** Prevalence of *Salmonella, Campylobacter*, and antibiotic resistance across various systems.

Source (Alphabetical)	*Salmonella*	*Campylobacter*	Antibiotic resistance
Alali et al., 2010	**Conventional:** 38.8 % in feces; 27.5 % in feed **Organic:** 6.5 % in feces; 5 % in feed	**N/A**	**Conventional:** 36.2 % *Salmonella* were resistant to streptomycin and 39.7 % resistant to AmStAxChCfFx **Organic:** <40 % resistance to tetracycline and streptomycin in *Salmonella*; 25 % *Salmonella* were resistant to streptomycin
Bailey and Cosby, 2005	**Free Range:** 31 % in chicken	**N/A**	**N/A**
Bailey et al., 2019	**N/A**	**Conventional:** Higher prevalence and population from feces and carcass rinse before antimicrobial interventions **Organic:** Lower prevalence and population from feces and carcass rinse before antimicrobial interventions; lower prevalence post water chill.	**Conventional:** Similar prevalence for AMR resistance between *Campylobacter* isolates as organic birds, except for tetracycline; less resistant to tetracycline resistance **Organic:** Similar prevalence of AMR resistance as conventional birds; more prevalent tetracycline resistance
Čermák and Skřivanová, 2016	**N/A**	**Pasture:** 28 % in chicken (cecal content)	**N/A**
Colles et al., 2008	**N/A**	**Free Range:** No connection **Wild Population:** No connection	**N/A**
Cui et al., 2005	**Conventional:** 44 % in chicken **Organic:** 61 % in chicken	**Conventional:** 74 % in chicken **Organic:** 76 % in chicken	**Conventional:** 54 % *Salmonella* were resistant to cephalothin-cefoxitin-ceftiofur **Organic:** 3.3 % *Salmonella* were resistant to cephalothin-cefoxitin-ceftiofur
Griggs et al., 2006	**N/A**	**N/A**	**Organic:** <40 % resistance to tetracycline and streptomycin in *Salmonella*
Hanning et al., 2010	**N/A**	**Pasture:** 30 % in farms, 75 % in retail carcasses, 100 % in processed chicken; rearing condition did not affect the prevalence but the genotypes	**N/A**
Heuer et al., 2001	**N/A**	**Conventional:** 36.7 % in chicken (cloaca) **Organic:** 100 % in chicken (cloaca)	**N/A**
Luangtongkum et al., 2006	**N/A**	**N/A**	**Conventional:** More *Campylobacter* isolates (< 2 %) resistant to fluoroquinolones **Organic:** Less *Campylobacter* isolates (< 2 %) resistant to fluoroquinolones
Melendez et al., 2010	**Pasture:** 25 % of environmental and feed/water samples in pasture farms **Retail Case:** 50 % in the retail carcasses	**N/A**	**Pasture:** All of the *Salmonella* isolates were resistant to sulfisoxazole and novobiocin; lower prevalence of antimicrobioal-resistant Salmonella than conventional birds.
Overbeke et al., 2006	**Conventional:** Similar **Organic:** Similar	**Conventional:** Lower prevalence **Organic:** Higher prevalence	**N/A**
Rothrock et al., 2016	**N/A**	**N/A**	**Pasture:** The MDR rate was 63.9 % for *Listeria*, 36 % for *Salmonella*, 12.7 % for *E. coli*, and 1.4 % for *Campylobacter*
Siemon et al., 2007	**Conventional:** 47 % positive farms; 30 % in feces **Pasture:** 33 % positive farms; 16 % in feces	**N/A**	**Conventional:** 69 % of MDR *Salmonella*; 0 % *Salmonella* resistant to ceftriazxone; **Pasture:** 11 % MDR *Salmonella*; 5 % *Salmonella* resistant to ceftriazxone;

### *Salmonella*

*Salmonella* prevalence is highly variable and can be found in all production types, including outdoor access (free-range and organic) and conventional systems. *Salmonella* was found in 61 % of organic and 44 % of conventional chicken carcasses from Maryland retail stores (United States, Cui et al., 2005). *Salmonella* was also isolated from 31 % of 135 free-range chicken carcasses and 25 % of 53 all-natural chicken carcasses (Georgia, United States, Bailey and Cosby, 2005), which far exceeded the allowable prevalence rate of 9.8 %, according to the USDA Performance Standard for *Salmonella* in raw poultry products (Federal Register, 2016). For pasture-raised poultry, *Salmonella* was detected in 25 % of environmental and feed/water samples at the farms and in 50 % in the retail carcasses (Arkansas, United States, Melendez et al., 2010). Taken together, these studies initially suggest that outdoor access farms have a higher risk for *Salmonella* than conventional farms, but other research shows contradicting results.

*Salmonella* prevalence in fecal and feed samples were higher for conventional farms than organic farms in North Carolina (38.8 % versus 6.5 % in feces and 27.5 % versus 5.0 % in feed; United States, Alali et al., 2010). Of 14 conventional farms and 19 pasture farms in Wisconsin and the Southeastern United States, conventional farms had numerically more positive *Salmonella* samples than the pasture farms, but no statistical significant differences were seen between the farm types (Siemon et al., 2007). Overbeke et al. (2006) reported no significant differences in the prevalence of *Salmonella* between organic (unspecified slow-growing breed) and conventional broilers by sampling hatching papers, overshoes on the farm, and gastrointestinal content of the birds in Belgium. Similarly, in a study that surveyed 27 retail stores in Louisiana, *Salmonella* prevalence was similar between organic (20.8 %) and conventional (22 %) chicken carcasses (United States, Lestari et al., 2009).

Overall, results are highly mixed in *Salmonella* prevalence among outdoor access versus conventional production systems. Prevalence likely depends on additional factors within and outside of the specific production facilities. For example, at the farm level, bird stressors such as heat, deprivation of feed or water, or high stocking density can alter the immune response and result in an increase of *Salmonella* (Shaji et al., 2023). At retail stores and the processing plants, microbiological control protocols may vary, such as sanitary dressing practices, antimicrobial applications, and/or temperature fluctuations during transportation of the product.

### *Campylobacter*

In general, the prevalence of *Campylobacter* in raw chicken is higher than *Salmonella* as *Campylobacter* continuously increases during the bird rearing phase. For example, *Campylobacter* was recovered in 76 % organic and 74 % conventional chicken carcasses in Maryland retail stores in the United States, which was higher than the recovery of *Salmonella* in the same carcasses *(*61 % of organic and 44 % of conventional chicken carcasses; Cui et al., 2005). *Campylobacter* also varies widely across production systems. *Campylobacter* was more prevalent in the feed and water of open-air housing systems compared to environmentally-controlled housing in Louisiana, although there were no differences between production systems in the live fast-growing Ross broilers through cloacal sampling (United States, Tangkham et al., 2016). Similarly, *Campylobacter* was most prevalent in broiler flocks with outdoor access (100 % of 22 organic flocks with an unspecified slow-growing breed), intermediate for indoor-only flocks with a lower stocking density than conventional (49.2 % of 59 extensive indoor flocks), and lowest in conventional indoor-only housing (36.7 % of 79 conventional flocks; Denmark, Heuer et al., 2001). Also in pastured broilers (slow-growing Hubbard JA757), there was a 28 % prevalence of *Campylobacter* compared to no *Campylobacter* detected in conventional broilers (Czech Republic, Čermák and Skřivanová, 2016). The prevalence of *Campylobacter* from pasture farms, a pasture flock processing facility, and organic retail carcasses were 30 %, 100 %, and 75 %, respectively (Arkansas, United States, Hanning et al., 2010). However, the authors of the previous study suggested that rearing conditions had no impact on the prevalence of *Campylobacter* as the rates were similar to conventional retail carcasses in other studies; yet a more diverse genotype of *Campylobacter* was found in pasture flocks than previously reported for conventional flocks (Hanning et al., 2010).

Other studies contradict the notion that outdoor access production is at a higher risk for *Campylobacter*. Wild birds are hypothesized to be a critical source of infection from a biosecurity perspective, yet a longitudinal survey of 64 free-range broiler flocks found no support for transmission due to the distinct differences in *Campylobacter* between broilers and wild bird species (starlings and geese) at the same sites (United Kingdom, Colles et al., 2008). Organic broilers reportedly have a higher prevelence of *Campylobacter* at slaughter, but this was attributed to the longer rearing period for the slow-growing breed rather than outdoor access (Belgium, Overbeke et al., 2006). Organic broilers had a lower prevalence and population of *Campylobacter* from feces and carcass rinses after feather picking compared to the conventional birds, which diminished further into processing due to the antimicrobial interventions used in plants (Midwest United States, Bailey et al., 2019). Thus, the prevalence of *Campylobacter* tends to be higher in flocks with outdoor access, but the prevalence can be highly variable, confounded with rearing time, and minimized during processing.

### *Antibiotic resistance*

Antibiotic resistance is a major concern in poultry production, encompassing resistance to individual antibiotics and multidrug resistance (**MDR**), where bacteria (e.g., *Salmonella* and *Campylobacter*) are resistant to multiple classes of antibiotics. Although antibiotic use is usually prohibited in free-range production, antibiotic-resistant microorganisms are still isolated from these systems (Cui et al., 2005; Griggs et al., 2006; Siemon et al., 2007; Melendez et al., 2010; Rothrock et al., 2016; Bailey et al., 2019). Antibiotic profiles can be dynamic and complex depending on the geographical region, farm, and flock. Rothrock et al. (2016) showed a large variance in the antibiotic-resistance profiles among bacteria when sampled at the farm and the processing plant for the same flock.

Some studies have found antibiotic resistance to be higher on conventional broiler farms than outdoor access broiler farms. *Salmonella* isolates from a conventional farm were resistant to ceftriaxone and 36.2 % were resistant to streptomycin, whereas the outdoor access organic farm resistance prevalence to streptomycin was 25 % (North Carolina, United States, Alali et al., 2010). Fewer *Campylobacter* isolates (< 2 %) from outdoor access organic poultry production were resistant to fluoroquinolones compared to 46 % of isolates from conventional broiler farms showing antimicrobial resistance (Ohio, United States, Luangtongkum et al., 2006).

However, outdoor access farms are not exempt from antimicrobial resistance. All of the *Salmonella* isolates from two pasture farms and retail carcasses were resistant to sulfisoxazole and novobiocin (Arkansas, Melendez et al., 2010). *Campylobacter* isolates from both conventional and organic broiler flocks were found to be similarly resistant to azithromycin, ciprofloxacin, clindamycin, erythromycin, florfenicol, gentamicin, nalidixic acid, and telithromycin at the processing plant, but tetracycline resistance was more prevalent for organic farms than conventional farms (Midwest United States, Bailey et al., 2019). This agrees with other studies in which *Campylobacter* isolated from antibiotic-free farms was most resistant to tetracycline (Minnesota, United States, Griggs et al., 2006; United States, Rothrock et al., 2016). Antimicrobial resistance can vary widely between studies though, where a similarly low rate of antimicrobial resistance in *Campylobacter* isolates was reported across organic, extensive indoor (i.e., lower stocking density than conventional), and conventional broiler flocks (Denmark, Heuer et al., 2001).

MDR is a critical aspect of antibiotic resistance, posing significant public health risks by limiting treatment options. The majority of research shows MDR as a higher risk on conventional farms compared to farms with outdoor access. MDR was reported in 69 % of *Salmonella* isolates from conventional farms and only 11 % from pasture farms in the Southeastern United States, with the predominant resistance to AmCSSuTeAx (ampicillin, chloramphenicol, streptomycin, sulfasoxazole, tetracycline, amoxicillin/clavalanic acid; Siemon et al., 2007). In the same study, no multidrug resistance was found in isolates from Wisconsin in either type of production system (Siemon et al., 2007). On conventional farms, 39.7 % of MDR (AmStAxChCfFx) was reported, while there was no MDR on organic farms (North Carolina, United States, Alali et al., 2010). The prevalence of *Salmonella* isolates resistant to cephalothin-cefoxitin-ceftiofur was 54 % from conventional chickens, whereas only 3.3 % of *Salmonella* isolates from organic chickens showed resistance (Maryland, United States, Cui et al., 2005). Moreover, *S.* Typhimurium isolated from conventional chickens were resistant to multiple antimicrobials, whereas the isolates from organic chickens were not (Cui et al., 2005). Outdoor access flocks can have varying levels of MDR though. Rothrock et al. (2016) surveyed the environmental and processing samples of 15 all-natural, antibiotic-free, pasture-raised broiler flocks (slow-growing Freedom Ranger and fast-growing Cornish Cross) from six farms in the southeastern United States, and they reported the MDR rate was 63.9 % for *Listeria*, 36 % for *Salmonella*, 12.7 % for *E. coli*, and 1.4 % for *Campylobacter*.

Overall, peer reviewed literature predominantly shows that antibiotic resistance, particularly MDR, is more prevalent in conventional production systems than in outdoor access production systems. Antibiotic-free practices appear to mitigate resistance levels by reducing selective pressure; however, resistant microorganisms are still isolated from these systems. These findings underscore the complexity of antibiotic resistance in poultry production and the need for comprehensive strategies to address both individual resistance and MDR.

### *Gut microbiome*

Poultry gut microbiota is generally influenced by diet (Apajalahti et al., 2004; Torok et al., 2011; Borda-Molina et al., 2016), environment (Xu et al., 2016; Rothrock and Locatelli, 2019), host genetics (Zhao et al., 2013), and age (Lu et al., 2003; Apajalahti et al., 2004; Videnska et al., 2014; Ocejo et al., 2019), with changes in the microbiome profile depending on the location in the intestine sampled (van der Wielen et al., 2002; Rehman et al., 2007; Yeoman et al., 2012; Ocejo et al., 2019). Access to the outdoor environment can lead to a more diverse microbiome in free-range birds due to the exposure to more heterogenous arrays of microbes from the soil, vegetation, insects, and wildlife (Shi et al., 2019).

Outdoor access areas differ in their unique local ecology, such that the same breed (slow-growing Freedom Ranger) of broilers fed the same diet differed in their microbiome richness and diversity between two different pasture sites (Georgia, United States, Rothrock and Locatelli, 2019). Free-range slow-growing Dagu chickens in China had a higher proportion of Bacteroidetes in the cecum but a lower Firmicutes/Bacteroidetes ratio than indoor-only reared chickens of the same breed (China, Xu et al., 2016). Although rearing conditions can impact the microbiome in live birds, the impact on the finished raw poultry products might be minimal. In a culture-based study on the microbiota of conventional and free-range chicken meat from Irish processing plants, it was suggested that the processing plants and storage determine the end-product microbiome (Marmion et al., 2023).

Since conventional production systems typically house fast-growing broilers fed with high-nutrient diets and outdoor access systems typically house slow-growing birds fed with low-nutrient diets, the differences in diets could confound differences in chicken gut microbiota related to housing environment. Lourenco et al. (2019a) showed that diet had an impact on cecal microbiota in pasture-raised broilers, as cecal operational taxonomic units increased after 12 weeks on a soy-free diet but there was no change for broilers fed a soybean diet (slow-growing Freedom Ranger, Georgia, United States). Additionally, fecal and carcass samples yielded richer microbiome diversity in the soy-free-fed birds than the soybean-fed birds at processing (Georgia, United States, Lourenco et al., 2019b).

Age is another confounding factor when comparing gut microbiota. Ocejo et al. (2019) reported a richer and more complex cecal microbiota in free-range raised birds (slow-growing Sasso-T451A) than in the conventionally raised birds (fast-growing Ross 308) in Spain, but this was attributed to age-related differences in the major taxonomic groups. Similarly, while dietary influence was observed at the early stage (up to 42 days) of free-range slow-growing Cobb 308 broilers fed different diets, age became a more dominant factor influencing the microbiota in the small intestine (Canada, Islam et al., 2019). Other bird characteristics, such as sex and genotype for body weight, affected 35.8 % of the bacterial species in the gut microbiota, despite rearing broilers under the same husbandry and dietary regimens (Virginia, United States, Zhao et al., 2013).

In summary, some evidence supports that broilers with outdoor access have a more diverse microbiome than broilers raised in conventional indoor-only housing. The relationship with outdoor access and microbiome diversity is shaped by an individual farm's local ecology. Diet and bird characteristics, such as age, breed, and sex, have confounding effects on the microbiome that cannot be attributed to the housing environment alone. Some of the studies cited did not include breed in their methodology, but given the impact of breed on the microbiome and consequences for other food safety measures, we recommend that future studies report bird breeds in their methodology.

## Meat quality

The type of rearing system used for live production can greatly impact the quality of meat for consumers after harvest (Marchewka et al., 2023). Broiler meat produced in alternative systems has become favorable for consumers because the products have value-added sensory qualities such as taste, flavor, and odor (Latter-Dubois, 2000; Castellini et al., 2002). In a recent review of the effect of husbandry factors on meat quality, Marchewka et al. (2023) explained the roles of diet, enrichment, stocking density, and genetics on meat quality and carcass characteristics. In the current review, we will primarily focus on the effect of outdoor access or housing/rearing system on meat quality (pH, color, water holding capacity, composition, texture, shelf life). Table 3 summarizes the main attributes evaluated for meat quality including pH, color, drip loss, cook loss, and water holding capacity based on production type. Table 4 summarizes composition, texture and sensory properties.

**Table 3:** Review of pH, color, Water Holding Capacity,based on housing type.

Source (Alphabetical)	pH	Color	WHC
Aksoy et al., 2021b	pH varied among rearing systems and fast versus slow-growing chickens	Free-range slow-growing (FRSG) birds were darker compared to the other treatments. Traditional free-range birds SG birds were less red compared to other treatments except for extensive indoor fast-growing (FG) birds. FRSG birds were yellower compared to extensive indoor/free-range FG chickens	% cook loss was greater in the summer compared to spring. Cook loss was only lower between free-range slow-growing birds and extensive indoor fast-growing birds
Berri et al., 2005	The fast grow line had a greater pH (6.45 at 15 m / 5.70 @ 24 h) than the Slow Grow line (6.32 @ 15 m / 5.66 @ 24h.)	Not different in lightness; SGL was redder and yellower	Fast-growing Line (FGL) had greater WHC due to the pH.
Brown et al., 2008	Slow-growing (5.85) and fast-growing (5.84) had greater pH than medium-growing (5.80) Organic (5.77) was not diff.	L) SGL and MGL were darker than FGL, organic was not diff. a) SLG was reddest, then FGL. MGL and Organic were the least red and not different from each other. b) MGL was yellower than the rest of the breeds.	NS
Castellini, 2002	Conventional had greater pH (5.98) than organic (5.80) at d 81	NS	WHC in organic was lower than in conventional breeds.
Chabault et al., 2012	pH for slow grow line on average was 6.61 at 15 m and 5.80 at 24h. NS between males and females	The coefficients of variation for the other meat quality traits varied considerably and were relatively small for lightness, moderate for yellowness, and quite high for redness.	The meat of females had higher drip loss
Cygan-Szczegielniak et al., 2019	pH_15_ and pH_24_ was higher in intensive system (IS) (indoor chickens with high stocking density) (6.32, 5.76) compared to semi-intensive systems (chickens given access to open-air, grassy pens) (6.10, 5.68)	Intensive system chickens were darker in color compared to semi-intensive chickens. Chickens from semi-intensive systems were yellower. Redness NS.	NS
Cygan-Szczegielniak et al., 2021	NS	NS	NS
da Silva et al., 2017	pH higher in industrial chickens (5.90) compared to free-range (5.75)	Lightness NS in breast meat. Industrial breast meat was redder but less yellow than free-range meat	N/A
Gálvez et al., 2020	pH in breast meat was lower in organic (5.74) and free-range (5.68) reared chickens compared to industrial (6.05) reared chickens. pH NS in drumstick meat.	Lightness NS in breast but darker in organic (O) drumstick compared to industrial (I) and range (R) reared chickens. Redness and yellowness from least to most was *I* < *O* < *R* in breast meat. Drumstick meat was redder in R and O chickens compared to I chickens. Yellowness was higher in R chicken drumstick meat.	N/A
Kim et al., 2020	At 9 d, pH was higher in conventionally grown chickens (6.34) compared to those reared in animal welfare systems (6.15)	NS	NS
Li et al., 2016	NS	N/A	NS
Mattioli et al., 2024	NS	NS	NS
Michalczuk et al., 2017	NS (Indoor at 5.77; Outdoor at 5.79)	NS in color	NS (Indoor at 11.87; outdoor at 12.98)
Özbek et al., 2020	pH in breast meat higher in free-range (5.71) and slate housing systems (5.69) compared to deep litter (5.54). pH NS in leg meat. Fast versus slow-growing genotypes NS.	NS in breast meat. Only deep litter housing systems were redder than the free-range and slats types. Genotype NS.	N/A
Sampels et al., 2021	NS	NS	% thaw and cook loss NS
Sarica et al., 2019	pH higher in some slow and medium-growing chickens. pH between birds indoor versus outdoor regardless of breed was higher in indoor (5.67 versus 5.62)	Different breeds of broilers showed differences in lightness, redness, and yellowness. Thigh meat in outdoor chickens were lighter compared to indoor chickens	N/A
Stadig et al., 2016	pH was lower in indoor reared chickens (5.73) versus free-range access (5.79) but the same for short-rotation coppice chickens (5.76)	Free-range chickens were darker and yellower compared to indoor chickens but NS between treatments for redness.	% Drip loss was lower in free-range chickens compared to indoor birds but % cook loss was NS
Tong et al., 2015	NS	Darkness and yellowness increased over longer treatments of outdoor access. Redness NS	NS
Woo-Ming et al., 2018	Fall chickens in hooped houses with and w/o pasture access had lower pH (5.62, 5.58) compared to spring chickens with all housing/production types and fall chickens with fixed houses with and w/o pasture access (range 5.66-5.95)	Lightness and yellowness NS. Only yellowness in the fall chickens from fixed houses with outdoor access were less red compared to chickens from hoop housing with pasture access	Cook loss was lower for spring chickens housed in hoops and pasture reared compared to all other factors
Yang et al., 2015	Δ pH was significantly larger for caged male birds (Δ0.28) and smaller for free-range (Δ0.26) and floor pens indoor reared (Δ0.25) male birds. Δ pH for females NS	N/A	% drip loss was lowest in male and female chickens in free-range (FR) systems compared indoor caged. Drip loss for FR was lower or same within a sex compared to floor pen indoor rearing systems

NS: Not significant; AF: farm with lower stocking density and ammonia than conventional, also provided foraging materials; SGL: slow-growing line; FGL: fast-growing line.

**Table 4:** Review of composition, texture, and sensory qualities based on housing system.

Source (Alphabetical)	Composition	Texture (Shear Values)	Sensory
Aksoy et al., 2021b	N/A	N/A	Consumers scored extensive indoor fast-growing (FG) and traditional free-range slow-growing (SG) meat the tenderest. Traditional free-range SG meat had the highest flavor score compared to other treatments except for free-range SG growing meat. Multiple treatments were overall acceptable to consumers
Berri et al., 2005	Higher fat in 6-FG	N/A	N/A
Brown et al., 2008	Organic chicken had less fat compared to conventional chicken.	Slow and medium-growing had tougher texture compared to fast-growing and organic, organic showed the most tender texture	All the breast meat samples were considered tender; however, meat from birds produced under standard conditions was tenderer than meat from birds produced under other systems. The juiciest meat came from the standard system and the least juicy from the organic system. There were NS differences in chicken flavor intensity.
Castellini, 2002	Organic birds had higher protein (19.47 %) and less fat (2.83 %) compared to conventional birds at d 81	NS between systems Tougher texture in older age.	The sensory quality of the breast muscle was better. The organic production system is a good alternative method, due to better welfare conditions and good quality of the carcass and meat. A negative aspect was the higher level of thiobarbituric acid-reactive substances in the muscles, probably due to greater physical activity.
Chabault et al., 2012	The lipid content of the breast meat was slightly higher for SGL.	Shear force has a relatively small variation between males and females.	N/A
Cygan-Szczegielniak et al., 2019	Protein was generally lower in the semi-intensive systems compared to the intensive systems. % fat NS.	Shear force was higher for intensive system birds.	N/A
Cygan-Szczegielniak et al., 2021	Protein and fat was lower in male chickens compared to female chickens both reared in organic systems.	Shear force was higher in male chickens compared to female chickens	N/A
da Silva et al., 2017	Ash NS in breast, thigh, and drumstick meat in industrial and free-range (FR) systems. Protein and lipid NS in breast meat. Protein was higher in FR thigh meat but NS in drumstick. Lipid was lower in FR drumstick meat	Shear force in free-range breast meat was higher	Small consumer test (*n* = 30) found overall impression, odor, flavor, texture, and juiciness NS. Color acceptability was higher in free-range meat
Evaris et al., 2021	Ash and protein were lower in breast meat from indoor chickens compared to outdoor chickens. Fat was higher in both indoor breast and thigh meat. Ash was lower in indoor thigh meat	N/A	N/A
Gálvez et al., 2020	Protein, ash, and fat varied between rearing systems and muscles. Histidine was the only free amino different. Several fatty acids and minerals were different between three rearing systems.	N/A	N/A
Kim et al., 2020	NS for fat, protein, and ash. Saturated fatty acids (FA) were higher in conventional birds and unsaturated FAs were lower in animal welfare birds.	Shear force was greater in chickens reared under animal welfare compared to conventional systems	N/A
Latter-Dubois, 2000	NS on Fat. There were significant differences in the breast meat between the different crossbreeds for protein	Results show that there were no significant differences between the crossbreeds of chickens for tenderness, juiciness, or flavor.	Statistically, there was no effect of the panel observed. No significant differences were found between individual chickens of each crossbreed. It was noted that there was non-significant variation between individual chickens within each crossbreed.
Mattioli et al., 2024	Breast and thigh meat was evaluated. NS for protein, ash, and fat. Sorghum enriched birds had > levels of α-tocopherol, total tocopherols, and thiols. Certain fatty acids increased or decreased between treatments	N/A	N/A
Michalczuk et al., 2017	Outdoor had more protein, and less fat compared to indoor chicken meat.	Outdoor chicken meat had a tougher texture than indoor ones.	N/A
Özbek et al., 2020	N/A	NS for housing and genotype alone	N/A
Sampels et al., 2021	Rearing system NS for % fat. Only a few fatty acids were significantly different between rearing systems	Rearing system had no effect on shear NS.	N/A
Stadig et al., 2016	NS for fat, protein, and ash. Free-range chicken had greater fatty acids compared to indoor chickens	NS	Panel found higher levels of fibrousness and lower levels of tenderness in breast meat in indoor and free-range chickens compared to short-rotation coppice (SRC) chickens. Juiciness was lower in indoors compared to SRC meat
Yang et al., 2015	N/A	Tougher texture in both male and female chickens raised in free-range systems compared to caged indoor systems.	N/A
Zidane et al., 2018	Protein was greatest in crested free-range compared to commercial broilers (CB) and two other phenotypes of chickens. Fat was the highest in CB compared to all others. Ash in CB was highest compared to two phenotypes of chickens	N/A	N/A

### pH

Literature reports that the ideal pH for chicken ranges from 5.8 to 6.3 post-harvest as this target range for pH results in higher water holding capacity, tenderness, and juiciness, translating to an improved consumer experience. If the pH is too low (< 5.3), it results in meat that can be pale, tough, and exudative (Smith and Northcutt, 2009; Adzitey and Nural, 2011; Chauhan and England, 2018). Whereas, a pH that is too high (> 6.3), results in meat characteristics that are darker, drier, and tougher (Adzitey and Nural, 2011; Loudon et al., 2018). Globally, there have been several studies with mixed results on pH post-harvest. In Italy, Castellini et al. (2002) investigated fast-growing Ross broiler carcass and meat quality traits at 56 and 81 days in conventional and organic production systems, where conventional birds were housed in an indoor-only pen (0.12 m^2^/ bird) and organic birds were housed in an indoor pen with the same stocking density and with access to a grass paddock (4 m^2^/bird). The researchers reported that both organic chicken breast and drumstick muscles had lower pH (breast: 5.75 at 56 d and 5.8 at 81 d; drumstick: 6.02 at 56 d and 6.10 at 81 day) compared to the corresponding muscles from conventional systems (breast: 5.96 at 56 d and 5.98 at 81 d; drumstick: 6.18 at 56 d and 6.25 at 81 d). These findings indicated that organic chicken breast and thighs had lower water holding capacity and may potentially taste tougher during consuming experiences. Lower pH was also measured in meat from slow-growing Sasso T451 organic chickens, which could have been due to less heat stress pre-slaughter or muscle fiber density and size differences in free-range rearing (Belgium, Stadig et al., 2016). Likewise, Brown et al. (2008) determined that the pH of chicken meat from a conventional indoor system (fast-growing Ross or Cobb breeds) was greater than chicken meat from the free-range and organic systems (slow-growing Hubbard breed) in England; the water holding capacity (**WHC**) was not significantly different among these treatments. However, Michalczuk et al. (2017) evaluated meat quality from Polish outdoor and indoor rearing systems using an experimental slow-growing hybrid breed, and they found no differences in pH of both breast and leg muscles between housing systems.

The effects of age, breed, and housing system on pH are confounded and difficult to disentangle. Berri et al. (2005) reported that pH 15 min post-mortem and the ultimate pH (**pHu**) values of breast and thigh muscle were lowest in 12-week-old slow-growing broilers and highest in 6-week-old fast-growing broilers housed indoor-only (Hubbard-ISA commercial crosses, France). These results agree with summarized findings from a recent review article, where slow-growing broilers from the French Label Rouge rearing system (provided with outdoor access and typically processed at 86 days of age) had a higher shear force value than conventional indoor-only fast-growing broilers typically processed at 35 to 42 days of age (Baéza et al., 2022). The review article attributed the tenderness differences to a lower pH and smaller diameter of muscle fibers in the Label Rouge rearing system, which could be linked to older age, but could also be linked to the outdoor rearing environment promoting more walking behavior and consequently more muscle activity than an indoor-only environment (Baéza et al., 2022). Similarly, meat from slow-growing Hubbard JA57 broilers had a lower pH than meat from fast-growing Ross 308 broilers in both indoor-only and free-range systems (Turkey, Aksoy et al., 2021b). Furthermore, season can influence pH, where the pH in meat from birds reared in the summer was higher than meat from birds reared in the spring (Aksoy et al., 2021b). In summary, it appears that meat from birds with outdoor access tends to have lower pH values than meat from indoor/conventional birds, which suggests conventional meat may be juicer and more tender compared to organic and/or free-range meat. However, some studies confound age, breed, and housing environment with pH metrics, which would be crucial to standardize moving forward to disentangle these effects.

### *Color*

Meat color is a critical parameter of meat quality that influences consumer choice during product selection (Pathare et al., 2013), and consumer preferences for meat color may vary depending on intercultural differences (Altmann et al., 2023). Meat color often depends on the myoglobin content, species, type of muscle, and age of the bird. Castellini et al. (2002) reported that breast and drumstick meat from fast-growing Ross broilers reared organically (including outdoor access) were brighter at 56 and 81 days of age than meat from the same breed housed indoor-only in Italy. Likewise, breast meat from free-range systems with a slow-growing Hubbard breed was brighter and less red, but did not differ in yellowness, when compared to breast meat from conventional, indoor-only systems with a fast-growing Ross or Cobb breed (England, Brown et al., 2008). However, Gálvez et al. (2020) reported no differences in the brightness of breast meat in conventional (fast-growing Ross 308), free-range (slow-growing Sasso XL-44), and organic-reared chickens (slow-growing Sasso T-44) in Spain, but observed more redness and yellowness in the free-range and organic breast meat.

Breed and housing system may have an interactive effect on meat color due to differences in muscle tissue structure, fat content, and pH (Mir et al., 2017). In Turkey, three housing systems were evaluated (free-range and indoor-only processed at 57 days, traditional free-range processed at 82 days of age) in combination with fast- (Ross 308) and slow-growing (Hubbard JA57) chickens (Aksoy et al., 2021b). The free-range slow-growing breed displayed a darker skin color compared to skin from the slow- and fast-growing chickens housed indoor-only. Both slow- and fast-growing broilers housed free-range had redder skin compared to broilers from indoor-only and traditional free-range housing. Additionally, meat from free-range fast-growing birds had darker color than meat from all other housing types, while meat from free-range slow-growing birds was yellower than free-range fast-growing birds. Moreover, the slow-growing birds in traditional free-range had less red meat than the same breed housed free-range and indoor-only. Taken together from one study (Aksoy et al., 2021b), these findings indicate complicated relationships with meat and skin color depending on breed and housing system. Seasonality can also affect skin color as the same researchers reported that chickens reared in the spring displayed darker, redder, and yellower skin compared to chickens reared in the summer, yet there was no difference between birds reared in the spring or summer (Aksoy et al., 2021b). However, in a different study conducted in Turkey, four slow-growing crossbreeds and two medium-growing crossbreeds were compared to commercial slow-growing Sasso broilers and commercial fast-growing Ross 308 broilers in indoor-only and free-range rearing systems (Sarica et al., 2019). The researchers found no significant differences in redness or yellowness in breast or thigh meat due to rearing systems, but thigh meat from outdoor chickens was lighter (Sarica et al., 2019). In summary, housing systems and breeds have mixed effects on the color of chicken meat or skin, which can be further exacerbated by season where birds with outdoor access may be moreso influenced than indoor-only birds. Ultimately these effects could impact consumer purchasing decisions.

### Cook Loss and Water Holding Capacity (WHC)

Cook loss and WHC are indicators used to determine further processed meat functionality and sensory profile, and WHC partially plays a role in meat juiciness and visual acceptability to consumers (Warner, 2023). Organic outdoor access chicken breast and drumsticks at 56 and 81 days of age both had greater cook loss in comparison to the conventional chicken meat (fast-growing Ross, Italy, Castellini et al., 2002). When comparing a 12-week-old slow-growing breed to a 6-week-old fast-growing breed, the 12-week-old birds had more breast drip loss and cooking loss compared to the 6-week-old birds (Hubbard-ISA commercial crosses, France, Berri et al., 2005), indicating that the fast-growing breed had better WHC for the final product quality. In contrast, indoor-only Ross 308 fast-growing chicken meat had greater cooking loss compared to free-range slow-growing Hubbard JA57 chicken meat (Turkey, Aksoy et al., 2021b). Within the slow-growing Hubbard JA57 broilers, chicken meat from traditional free-range (i.e., harvested at 82 days) had the lowest thaw loss compared to free-range (i.e., harvested at 57 days) and indoor-only housing (Turkey, Aksoy et al., 2021b). However, multiple studies have found no effect of housing type or breed on cook loss and WHC. Sampels et al. (2021) reported percent cook loss was not different in two different slow-growing genotypes (Rowan Ranger and Hubbard CYJA57) reared with outdoor access or indoor-only in Sweden. These results agree with Michalczuk et al. (2017), who reported cook loss and WHC were not different among breast and leg muscles from an experimental slow-growing hybrid breed of chickens reared with outdoor access and indoor-only in Poland. Also in Poland, researchers found that outdoor access did not affect WHC for Ross 308 fast-growing broilers (Cygan-Szczegielniak et al., 2021). Similarly in the United States, fast-growing Cobb chickens from different growing seasons (fall versus spring), housing type (hoop versus fixed), and pasture access (access or none) did not show significant changes in the WHC between production systems (Arkansas, Woo-Ming et al., 2018). The WHC of chicken meat from fast-growing Cobb or Ross breeds and a slow-growing Hubbard breed was not significantly different amongst conventional, free-range, and organic housing systems (England, Brown et al., 2008), and medium-growing Lingnanhuang broilers also did not differ in the WHC of the breast meat 90 days of age when reared in caged, indoor-floor, and free-range systems (China, Li et al., 2016). Cook loss and WHC results from literature are complicated and mixed but tend to show there are no differences between housing treatments and differences are more pronounced between breeds, with some studies showing fast-growing birds having lower cook loss and better WHC.

### *Texture*

Texture is a key characteristic to poultry products as it can determine product uses and is summarized along with composition and sensory profiles in Table 4. Texture can be measured using analytical instruments alone or in combination with sensory evaluations to predict consumer perceptions (Barbut, 2015). Shear tests are commonly used to evaluate toughness and tenderness in poultry products with the Warner Bratzler shear test (shear force), where greater shear values indicate tougher pieces of meat (Barbut, 2015). Rearing system, age, and breed can affect tenderness (Owens and Meullenet, 2010). Meat from older fast-growing Ross chickens, regardless of rearing type, had a tougher texture in comparison to the younger-aged meat (Italy, Castellini et al., 2002). The same researchers also determined that the organic chicken breast and drumsticks had greater shear values compared to indoor-only rearing, which also increased with age (Castellini et al., 2002). Similarly, when comparing an experimental slow-growing breed in different rearing systems, chicken breast meat from birds with outdoor access was tougher than the meat from the same breed reared indoors (Poland, Michalczuk et al., 2017). Tougher thigh meat was also reported for a slow-growing native Chinese chicken breed in a free-range system compared to chicken meat from birds caged indoors (China, Yang et al., 2015). Yet, when investigating the effect of the age of outdoor access (14, 21, 38, and 35 days of age) until slaughter at 56 days of age, thigh meat from the treatments were not significantly different for a different slow-growing native Chinese chicken breed (China, Tong et al., 2015). The researchers hypothesized that the level of exercise may not have reached a critical value to affect tenderness (Tong et al., 2015). In contrast, a study that compared housing systems (free-range, slat, and deep litter) reported no significant differences in toughness for breast and leg meat from fast- and slow-growing broilers (breed not specified); however, the variation in tenderness was attributed to the age of the bird prior to slaughtering (56 days of age, Turkey, Özbek et al., 2020). In summary, age, breed, and housing system affect the texture of the chicken meat. Slow-growing breeds, outdoor access, and older birds tend to have tougher meat compared to fast-growing breeds, indoor-only housing, and/or younger chicken meat.

### *Composition*

Composition of the muscle/meat of broiler chickens is often linked to water holding capacity and texture. Housing systems and breed contribute to varying protein, fat, and ash composition profiles (Marchewka et al., 2023). Breast meat from organic (slow-growing Sasso T-44) and free-range (slow-growing Sasso XL-44) chickens contained higher levels of protein compared to conventionally reared chickens (Ross 308; Spain, Gálvez et al., 2020). Free-range and organic chickens are hypothesized to have higher protein composition due to the ability to exercise in the outdoor area (Gálvez et al., 2020). When housed only indoors at the same stocking density, three slow-growing breeds (Plumage, Crested, and Tarsus) had higher levels of protein and fat compared to an unspecified commercial broiler breed, but fat and ash content varied (Algeria, Zidane et al., 2018). However, another study reported that protein content in the breast and thigh did not differ between indoor-only and organic rearing systems for a fast-growing Ross breed, but organic drumsticks had greater ash levels compared to indoor-only (Italy, Castellini et al., 2002). When rearing an experimental slow-growing breed with outdoor access or indoor-only, there were no differences in the protein, fat, moisture, and ash content in both breast and leg muscles (Poland, Michalczuk et al., 2017).

Abdominal fat is inversely related to protein in which a lower percentage of fat yields a higher percentage of protein. At ages 56 days and 81 days, abdominal fat and the percentage of fat in the breast and drumsticks were lower for organic fast-growing Ross broilers compared to the same breed reared indoor-only (Italy, Castellini et al., 2002). Likewise, Berri et al. (2005) reported that the breast meat of 6-week-old fast-growing birds had less protein, more fat, and contained muscle fibers with larger diameters than those of 8 week-old medium-growing and 12-week-old slow-growing birds (Hubbard-ISA commercial crosses, France). Similarly, slow-growing Rhode Island Red chickens raised outdoors had greater protein content in breast muscles and greater ash content and less fat in both breast and leg muscles compared to the same breed of chickens raised indoors (Mexico, Evaris et al., 2021). Therefore, outdoor access may lower fat content and increase protein levels, and fast-growing breeds could have less protein and higher fat compared to slow-growing breeds.

Rearing system may modify the health-promoting properties of meat (Michalczuk et al., 2017). Outdoor access rearing for a slow-growing experimental breed increased vitamin E levels, lowered saturated fatty acids, and increased n-3 polyunsaturated fatty acids in both breast and leg muscles compared to the same breed reared indoors (France, Michalczuk et al., 2017). Sampels et al. (2021) also observed that slow-growing Rowan Ranger and Hubbard CYJA57 broilers with outdoor access had higher levels of polyunsaturated fatty acids compared to the same breeds reared indoors in Sweden. The elevated concentrations of polyunsaturated fatty acids in free-range chickens may provide health benefits for consumers as this group of fatty acids has been linked to lowering the risk of cardiovascular diseases (Baum et al., 2012). Congruently, free-range and organically reared slow-growing breeds (Sasso XL-44 and Sasso T-44) broilers tend to have lower levels of cholesterol compared to fast-growing Ross 308 chickens reared indoors (Spain, Gálvez et al., 2020). However, one study reported no differences between fast-growing Cobb broilers given access to pasture or raised indoors (Arkansas, United States, Woo-Ming et al., 2018). The researchers hypothesized that the pasture used in the study may not have been established for birds to forage or that the fast-growing Cobb breed did not forage as much as slow-growing breeds from other studies (Woo-Ming et al., 2018). In summary, slow-growing breeds and/or outdoor access housing tend to have more protein and less fat content in most studies, which could be attributed to bird behavior promoted by the outdoor space and/or breed.

### *Sensory analysis*

Descriptive and consumer sensory analyses provide insight into how rearing systems affect sensory properties. Meat from broilers with outdoor access is commonly ranked with better sensory properties than meat from broilers reared indoors. In a trained taste panel, breast meat from birds raised under short-rotation coppiced willow tree systems were rated as more tender and less fibrous compared to breast meat birds raised under indoor housing and free-range with artificial shelters (slow-growing Sasso T451, Belgium, Stadig et al., 2016). Similarly, breast meat from slow-growing Hubbard JA57 broilers reared free-range was generally evaluated with better flavor than fast-growing Ross 308 broilers reared indoors (Turkey, Aksoy et al., 2021b). Although meat from a slow-growing RedBro cross genotype did not differ in tenderness, juiciness, and flavor with or without outdoor access, the outdoor access meat had a greater overall consumer acceptability compared to meat from indoor-only housing (Portugal, Ponte et al., 2005). Consumer sensory results suggest tradeoffs for certain meat sensory qualities with different durations of outdoor access (Turkey, Zaid et al., 2020). Considering the different durations that fast-growing Ross 308 broilers had outdoor access (21 d, 28 d, or 35 d as the age of outdoor access, or indoor-only), consumers rated meat with earlier outdoor access (at day 21) with better taste, flavor, and juiciness followed by outdoor access starting at day 28 and 35, with indoor-only rearing ranking last. However, meat from indoor-only birds was classified as more tender than meat from birds with outdoor access at day 35, followed by access at day 28 and 21. Meat from a slow-growing Hubbard breed in organic housing was also considered less tender and juicy than meat from Ross or Cobb fast-growing breeds reared indoors (England, Brown et al., 2008). Furthermore, based on flavor and overall liking of the meat, the meat from the indoor rearing was most preferred, while meat from organic systems was the least preferred, with free-range and maize-fed production systems ranking intermediate in preference (England, Brown et al., 2008). While more tender meat appears to be associated with indoor rearing or production systems with fewer opportunities for bird movement, flavor and juiciness seem to improve with outdoor access, but findings are inconsistent. Therefore, additional sensory analyses would help to identify potential organoleptic differences between different production systems.

### *Shelf Life*

Microbiological and oxidative stability are factors that can influence the shelf life of fresh poultry meat (Kong and Singh, 2016). Chicken meat from free-range poultry may have a shorter shelf-life than meat from commercially raised chickens due to having higher total aerobic plate counts. Alvarado et al. (2005) proposed that smaller processors in the United States may lack sufficient microbiological controls to keep the microbial load lower. However, in a study that investigated chickens reared under conventional indoor-only or an “animal welfare system” (i.e., indoor-only with a lower stocking density than conventional and additional substrates for pecking), chicken breasts from the animal welfare system had lower total aerobic plate counts after 9 d stored at 2 ± 2°C (fast-growing Arbor Acres, South Korea, Kim et al., 2020). The lower microbial load was potentially due to chickens from the animal welfare system having more room to roam, which may decrease the likelihood of microorganisms spreading between birds (Kim et al., 2020). Although the birds in that study did not have outdoor access, rearing birds with more space to move in outdoor access, coupled with proper sanitation procedures at processing, may reduce the initial microbial load and thus improve microbial shelf life. However, it is worth noting that chickens can come into contact with more vehicles that may carry microbes in an outdoor environment (e.g., insects, feces from other animal species; da Silva et al., 2017). Interestingly, da Silva et al. (2017) saw no differences in the mesophilic bacteria in commercial (fast-growing Cobb and Ross) versus free-range (slow-growing Gigante Negro, Isa Label, and Paraíso Pedrês or Label Rouge) chickens in Brazil.

Chicken meat contains unsaturated fatty acids that are susceptible to oxidation, which leads to off-flavor development such as “warmed over flavor” (Jayasena et al., 2013). The absence or reduction of α-tocopherol in the meat is believed to cause flavor deterioration as this compound may serve as an antioxidant to the fatty acids (Jayasena et al., 2013). A study in Italy investigated four slow-growing chicken genotypes (Red JA57, Naked Neck, Lohmann Dual meat-type, and Robusta Maculata x Sasso) that were provided outdoor access and either a sorghum-enriched diet or control diet, and they found that sorghum-enriched diets increased α-tocopherol content (Mattioli et al., 2024). These findings indicate that access to certain plant/grass species increased Vitamin E content, which can potentially enhance the shelf life of the meat.

In summary, the literature on meat quality from outdoor access varies due to experiment-specific methodology, and there is not a consistent pattern for different meat quality parameters of pH, meat color, WHC, drip/cook loss, composition, texture, and sensory properties based on production systems. There are mixed findings about outdoor access systems on meat quality, where pH levels may not be consistent, leading to drip/cook loss being inconsistent. Chicken meat color from outdoor access tends to be darker and tougher with less fat, while conventional systems produce meat to be lighter in color, more tender texture, and with more fat. However, there is also literature demonstrating no differences in these parameters. Some literature reported differences in protein, fat, and ash content, while others may not depending on the age, breed, or diet of the broilers being raised in outdoor versus conventional systems. Additionally, some literature suggests that outdoor access may lower fat content and increase protein levels, and fast-growing breeds could result in composition with less protein and higher fat.

## Conclusion

Shifting to outdoor access production offers benefits for animal welfare, food safety, and meat quality. Typically, welfare-related health outcomes (i.e., footpad dermatitis, mortality, and lameness), antimicrobial resistance, microbiome complexity, and some components of meat quality are improved with outdoor access compared to conventional housing. However, there is a high degree of variability in outdoor access production, such as climate, range quality, bird characteristics, and farm management. This variation results in highly variable range use by birds, a broad diversity of endo- and ectoparasitic species that can infect broilers, mixed results for the prevalences of *Salmonella* and *Campylobacter* between conventional and outdoor access farms, and challenges with texture and moisture of meat products. There may be opportunities to standardize outdoor access management to improve these outcomes and yield more consistency. We cannot eliminate the potential that the variable outcomes we observed in the literature may be due to methodological differences across studies. However, the current literature is consistent with the hypothesis that environmental and genetic variation mediate the effects of outdoor access on animal welfare and other outcomes. Further development of outdoor access management schemes may need to better account for environmental and genetic variation to drive improvement in animal welfare and other outcomes. It is worth noting that the majority of the research cited in this review was conducted outside of the United States. Geography and regional climates contribute to different outcomes, and some of the breeds used in previous research are not commercially available in the United States. Therefore, it is crucial to conduct more outdoor access broiler research in the United States to understand the full picture of outdoor access for relevant animal, microbial, and food product outcomes.

## Declaration of competing interests

The authors declare the following financial interests/personal relationships which may be considered as potential competing interests:

A. N. Pullin, Y. L. Campbell, and L. L. Walker report financial support was provided by General Mills, Inc., and they report a relationship with General Mills, Inc. that includes funding grants.
